# The number of physicians is related to the number of nighttime emergency surgeries in Japan: An ecological study

**DOI:** 10.1371/journal.pone.0278517

**Published:** 2022-12-01

**Authors:** Yusuke Yamadori, Tomohiro Hirao, Kanae Kanda, Gotaro Shirakami

**Affiliations:** 1 Department of Anesthesiology, Faculty of Medicine, Kagawa University, Miki-cho, Kagawa, Japan; 2 Department of Anesthesiology, Takamatsu Red Cross Hospital, Takamatsu, Kagawa, Japan; 3 Department of Public Health, Faculty of Medicine, Kagawa University, Miki-cho, Kagawa, Japan; Bach Mai Hospital, VIET NAM

## Abstract

**Purpose:**

Increasing the number of physicians per population may improve the quality of medical services, but there are few reports on this aspect in the field of surgery. This study aimed to examine whether the number of physicians is associated with the number of nighttime emergency surgeries, which may be one of the indicators of the quality of medical services in the field of surgery.

**Methods:**

This was a prefecture-based ecological study utilizing open data from Japanese government surveys conducted between 2015 and 2019. The relationship between the number of physicians and the number of nighttime emergency surgeries in 47 prefectures of Japan was evaluated by correlation analysis and panel data regression analysis. The correlation analysis was conducted between the number of physicians per 100,000 population and the number of nighttime emergency surgeries per 100,000 population per year in each prefecture in Japan. In the panel data regression analysis, panel data of the prefectures in Japan from 2015 to 2019 were created. We evaluated whether the number of physicians was related to the number of nighttime emergency surgeries, independent of the number of acute care beds per 100,000 population, population density, and the elderly population ratio.

**Results:**

From the correlation analysis, the correlation coefficient between the number of physicians per 100,000 population and the number of nighttime emergency surgeries per 100,000 population was 0.533 (P < 0.001). In the panel data regression analysis, there was a significant association between the number of physicians per 100,000 population and the number of nighttime emergency surgeries per 100,000 population (P < 0.001). The regression coefficient (95% confidence interval) for the number of physicians per 100,000 population was 0.246 (0.113–0.378).

**Conclusion:**

The number of physicians is associated with the number of nighttime emergency surgeries.

## Introduction

Presently, shortage and maldistribution of physicians in Japan are topics of discussion [1–6]. In 2008, the Japanese government changed its policy to increase the number of physicians [7], and in the recent years, the number of physicians increased by approximately 5,000 annually. However, the number of physicians per 1,000 population in Japan (2.5) is very small compared to the average for the Organization for Economic Cooperation and Development (OECD) member countries (3.6) [8]. Owing to the uneven distribution of physicians, medical supply varies greatly from region to region in Japan [3–6].

Several reports have suggested an association between the number of physicians per population and the quality of medical services in the field of primary care [9–12]. However, there are not many reports related to the field of surgery [13, 14]. Delay in emergency surgery is associated with adverse outcomes, such as in-hospital death, prolonged hospital stay, and increased hospitalization costs [15–17]. Sufficient human resources are required to perform timely emergency surgery. As it is more difficult to secure medical staff at night than during the day, the number of nighttime emergency surgeries being performed may indicate the quality of medical services in the field of surgery.

To our best knowledge, no studies to date have examined the relationship between the distribution of physicians and the nighttime emergency surgeries performed. Therefore, this study aimed to examine whether the number of physicians is associated with the number of nighttime emergency surgeries.

## Methods

This study used a prefecture-based ecological study design. All analyzed data were open data from Japanese government surveys [18–22] conducted between 2015 and 2019. The need for ethical review was waived by the Ethics Committee of the Kagawa University Faculty of Medicine because this study was not conducted on human participants and all information used were aggregate data. The objective variable was the number of nighttime emergency surgeries per 100,000 population per year, while the explanatory variable was the number of physicians per 100,000 population. The number of acute care beds per 100,000 population, population density, and the elderly population ratio were included as adjustment factors. The number of physicians and the number of acute care beds were included in the analysis as factors of medical supply; population density was included as a geographical factor (access to hospitals); and the elderly population ratio was included as an age structural factor.

The number of nighttime emergency surgeries was defined as the number of additional fees for nighttime anesthesia (for emergency inpatient surgeries starting from 22:00 to 05:59 the next day) per 100,000 population. The National Database of Health Insurance Claims and Specific Health Checkups of Japan (NDB) [18] open data were used for the number of additional fees for nighttime anesthesia. The NDB is the database of medical claims constructed by the Ministry of Health, Labor and Welfare (MHLW). The NDB data include nearly all the medical claims data in Japan, a country that provides medical insurance under a universal health insurance system. Physicians were included if they engaged in medical services in any specialty and their number was based on the Statistics of Physicians, Dentists and Pharmacists [19] published by the MHLW. The surveys are conducted every 2 years, and in the years without a survey, the number of physicians was estimated from the data of the previous and following years. The number of acute care beds was defined based on the number of beds in acute care hospitals with ≥ 100 beds. For the number of acute care beds, the data from the Survey of Medical Institutions [20] published by the MHLW were used. The area of prefectures was based on the Municipalities Area Statistics of Japan [21] published by the Geospatial Information Authority of Japan. To obtain the data of the overall population according to the age group, we used the Population Estimates [22] published by the Ministry of Internal Affairs and Communications. The elderly ratio was defined as the ratio of the population aged ≥ 65 years to the overall population.

The relationship between the number of physicians and the number of nighttime emergency surgeries in 47 prefectures of Japan was evaluated by correlation and panel data regression analyses. Correlation analysis was conducted to determine the relationship between the number of physicians per 100,000 population and the number of nighttime emergency surgeries per 100,000 population per year in each prefecture in Japan. The correlation analysis were based on the average values of all variables for each prefecture from 2015 to 2019. In panel data regression analysis, panel data (a data format that combines time-series and cross-section data) of the prefectures in Japan from 2015 to 2019 were created. We evaluated whether the fixed or random effects model was appropriate by performing the Hausman test, and the analysis was performed using the appropriate model. We evaluated whether the number of physicians was related to the number of nighttime emergency surgeries independently of the covariates, and calculated the regression coefficients.

Statistical significance was set at P < 0.01, and all P-values reported were two-sided. Statistical analyses were conducted using Stata 17.0 software (Stata Corp., College Station, TX, USA). ArcGIS 10.4.1 for Desktop (ESRI Inc., Redlands, CA, USA) was used to create the distribution maps of the number of physicians and nighttime emergency surgeries. Digital National Land Information [23] published by the Geospatial Information Authority of Japan and Prefecture level boundaries of Japan [24] from ESRI Japan (ESRI Japan Corp., Tokyo, Japan) were used for the creation of the blank map and prefectural borders.

## Results

**Fig 1:**
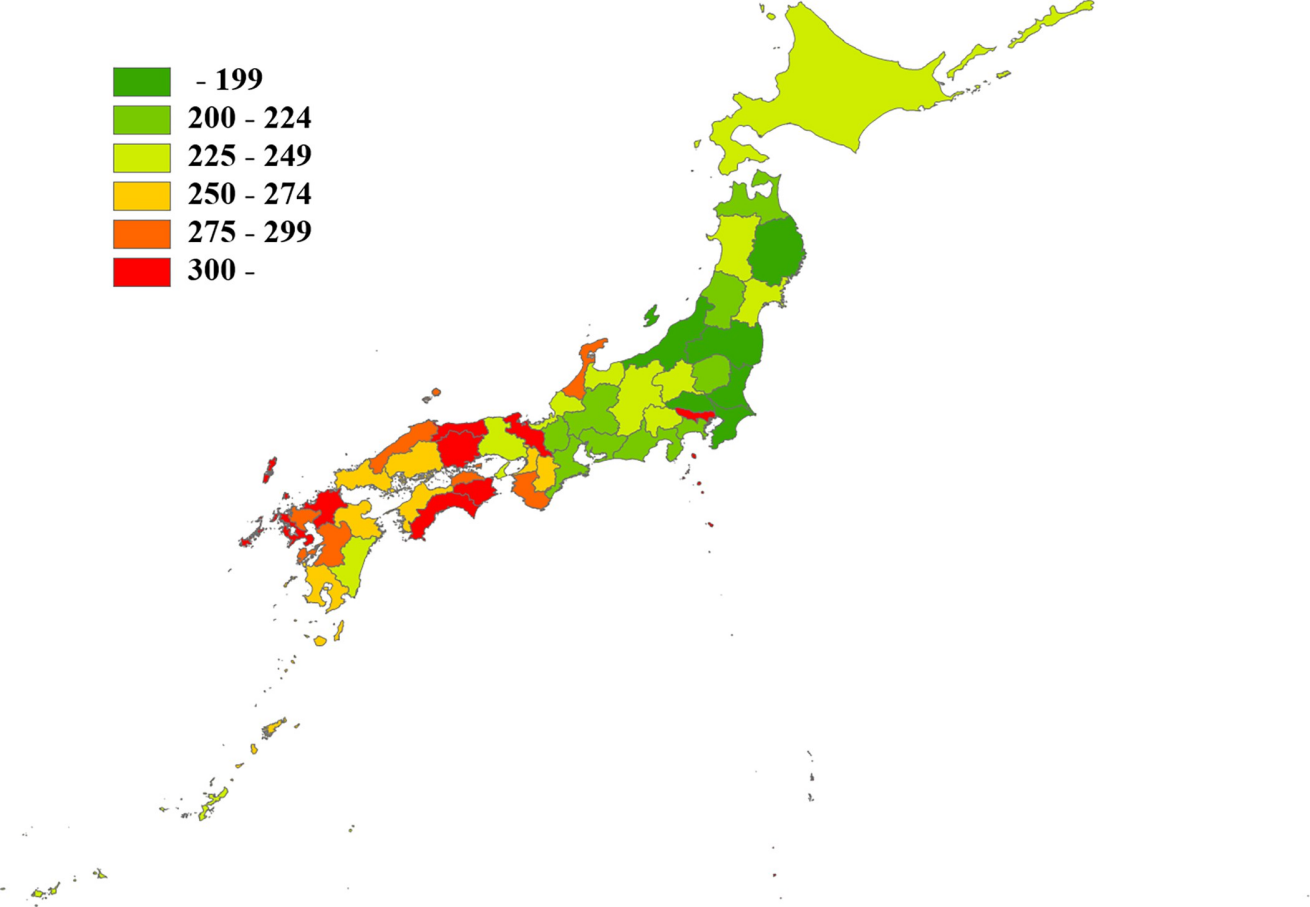
Number of physicians per prefecture. The presented values are the average numbers of physicians per 100,000 population in each prefecture (2015–2019).

**Fig 2:**
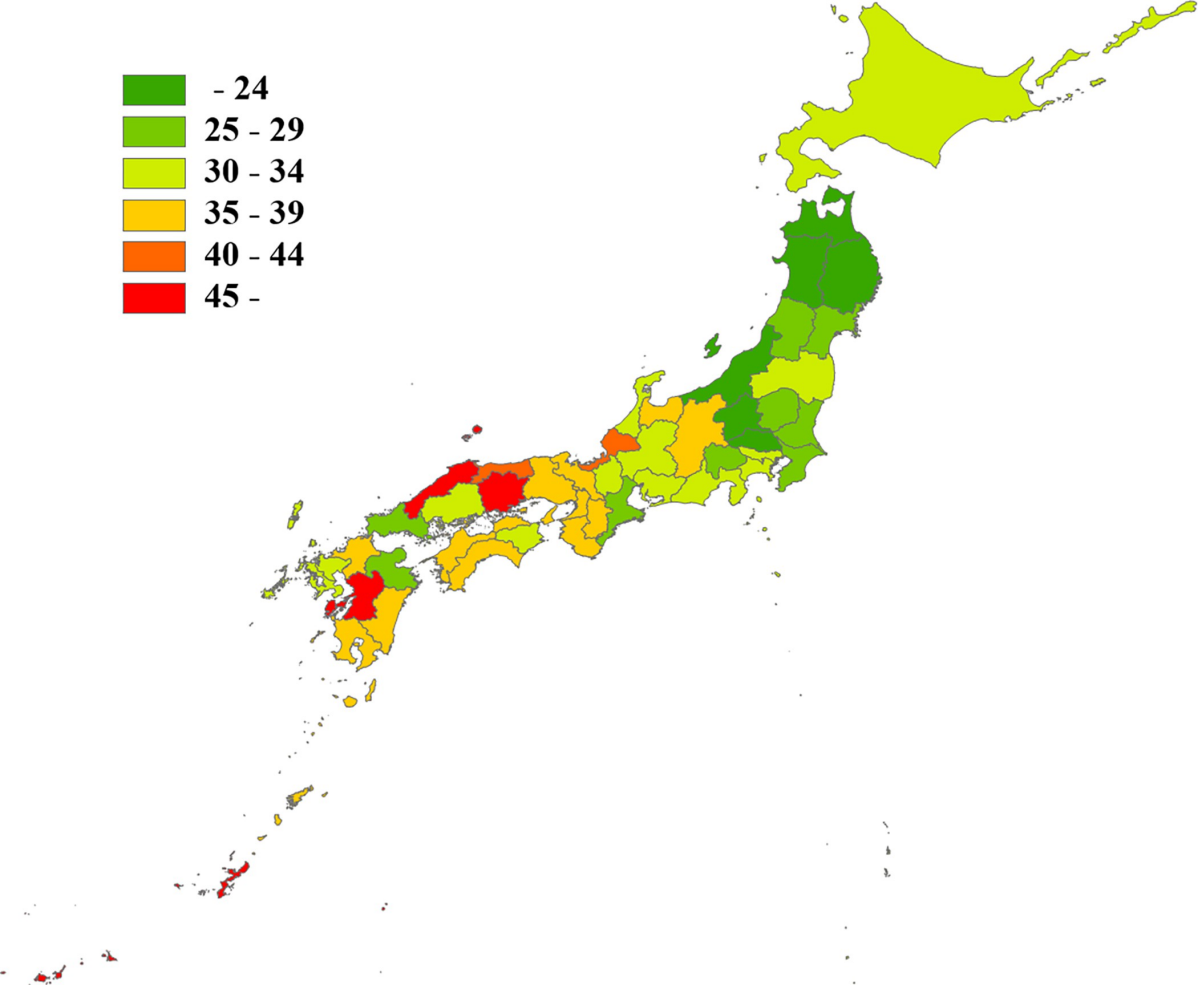
Number of nighttime emergency surgeries in each prefecture. The presented values are the average numbers of nighttime emergency surgeries per 100,000 population per year in each prefecture (2015–2019).

Figs 1 and 2 show the average number of physicians and nighttime emergency surgeries per 100,000 population in each prefecture in Japan from 2015 to 2019. The national average number of the physicians and nighttime emergency surgeries from 2015 to 2019 per 100,000 population were 243.7 and 32.9 per year, respectively. Table 1 shows the breakdown of the average number of physicians by medical specialty from 2015 to 2019.

**Table 1:** Breakdown of the physicians by medical specialty.

Total number of physicians	Internists	Surgeons	Anesthesiologists	Emergency physicians	Residents	Others
251.7	114.3	83.3	7.9	3.0	14.1	29.1

The presented values indicate the average numbers of physicians per 100,000 population in each medical specialty in Japan (2015–2019).

**Fig 3:**
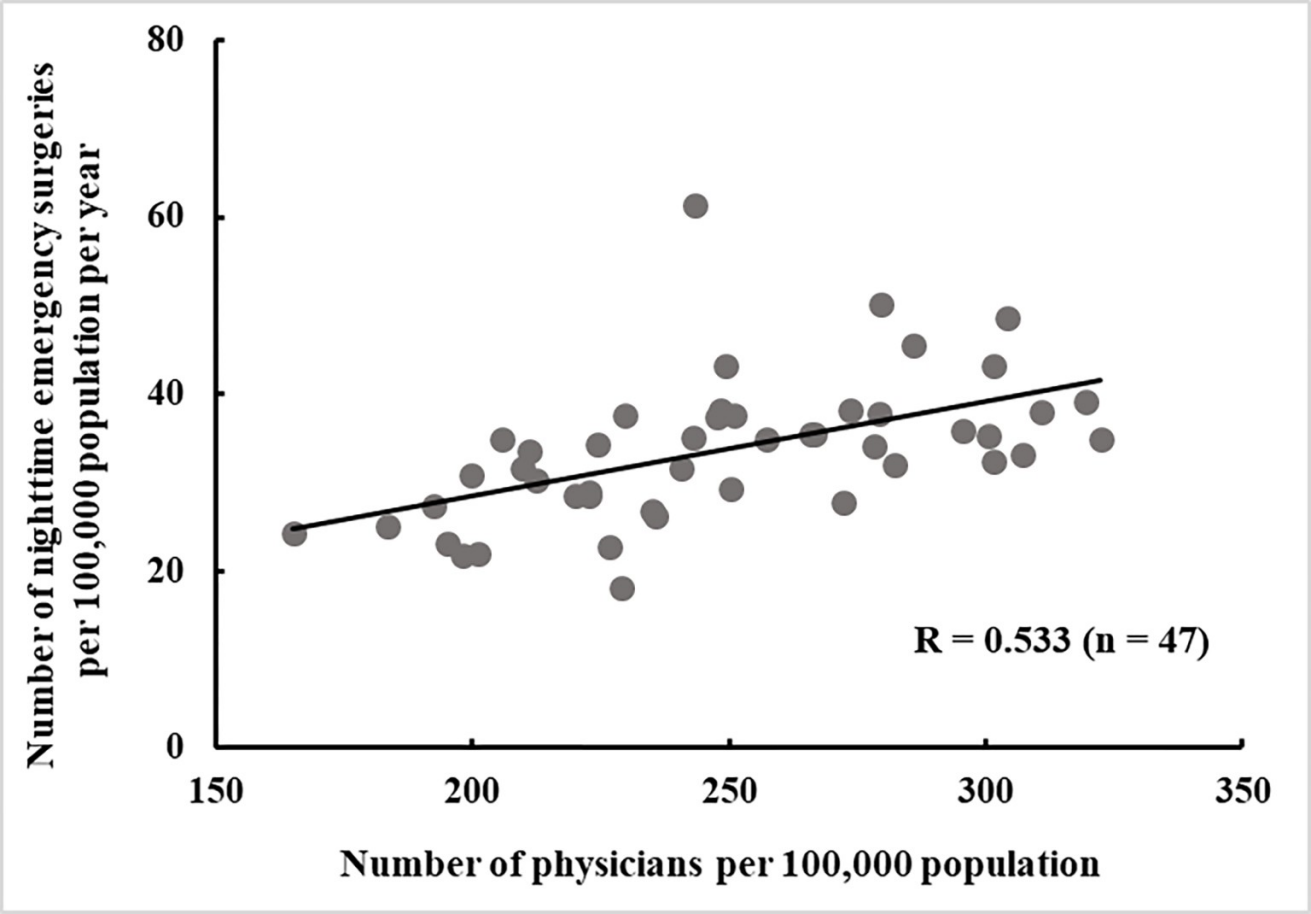
Correlation coefficient between the number of physicians and nighttime emergency surgeries. Correlation analysis coefficient between the number of physicians per 100,000 population and the number of nighttime emergency surgeries per 100,000 population per year (n = 47). R, correlation coefficient.

From the correlation analysis, the correlation coefficient between the number of physicians and number of nighttime emergency surgeries per 100,000 population was 0.533 (P < 0.001) (Fig 3).

In the panel data regression analysis, a fixed effects model was employed because the Hausman test detected bias in the estimates in the random effects model (P < 0.001). There was a significant association between the number of physicians and the number of nighttime emergency surgeries per 100,000 population (P < 0.001) (Table 2). The regression coefficient (95% confidence interval) for the number of physicians per 100,000 population was 0.246 (0.113–0.378). Other adjustment factors were not related to the number of nighttime emergency surgeries.

**Table 2:** Panel data regression analysis to estimate the influence of the number of physicians on the number of nighttime emergency surgeries per 100,000 population.

Variables	Regression coefficient	P value
	(95% confidence interval)	
Number of physicians per 100,000 population	0.246 (0.113–0.378)	< 0.001
Number of acute care beds per 100,000 population	0.006 (-0.285–0.041)	0.716
Population density (/km^2^)	-0.008 (-0.389–0.022)	0.588
Elderly population ratio (%)	-0.793 (-1.745–0.159)	0.862

Panel data regression analysis with a fixed effect model from 2015 to 2019 (n = 47).

## Discussion

The study results indicate that the number of physicians was associated with the number of nighttime emergency surgeries, which may be one of the indicators of the quality of medical services in the field of surgery. To our best knowledge, this is the first study to demonstrate a relationship between the number of physicians and the number of nighttime emergency surgeries performed. The maldistribution of physicians and the appropriate number of physicians have been discussed previously [3–6, 9–14, 25–27]. However, most of these studies were conducted in the field of primary care, and evidence in the field of acute care is still lacking. Further research is needed in the future.

In Japan, there are differences in the regional provision of health care services. The results of this study showed that the prefecture with the highest number of physicians per population had approximately twice as many physicians as the prefecture with the lowest (322.3 vs. 165.0 per 100,000 population) (Figs 1 and 3). The prefecture with the highest number of nighttime emergency surgeries per population had approximately three times as many nighttime emergency surgeries as the prefecture with the lowest number (61.5 vs. 18.1 per 100,000 population per year) (Figs 2 and 3). Okinawa Prefecture had the highest number of nighttime emergency surgeries per population, which was an outlier in the correlation analysis (Fig 3). Okinawa Prefecture is the southernmost and westernmost prefecture in Japan. It covers a vast medical area owing to its large sea area and many remote islands, and its medical system is very different from those of other prefectures. The correlation coefficient between the number of physicians per population and the number of nighttime emergency surgeries per population in Japan was 0.533 (P < 0.001), but when Okinawa Prefecture was excluded, the value was 0.634 (P < 0.001).

In the panel data regression analysis, there was a significant association between the number of physicians and the number of nighttime emergency surgeries, even after adjusting for the number of acute care beds, population density, and elderly ratio. The regression coefficient (95% confidence interval) for the number of physicians per 100,000 population and the number of nighttime emergency surgeries per 100,000 population per year was 0.246 (0.113–0.378) (P < 0.001); however, with Okinawa Prefecture excluded, it was 0.272 (0.132–0.413) (P < 0.001).

The factor most likely contributing to the study results is the uneven distribution of human resources involved in nighttime care. Sufficient human resources are needed to provide timely surgery at night. The large number of physicians per population may enable timely surgery with a positive impact on health care. It may be necessary to make policies that not only increase the number of physicians but also promote the availability of an appropriate number of physicians per population and improve the maldistribution of physicians.

The strength of this study is that it included nearly all the domestic data on nighttime emergency surgeries from the NDB open data. NDB includes nearly all the medical claims data in Japan under the universal health insurance system. Moreover, the panel data analysis is a time series data analysis, and the estimation accuracy can be improved by increasing the amount of data.

This study also had some limitations. First, this was an ecological study [28] using aggregate data. There was a relationship between the number of physicians and nighttime emergency surgeries, but it remains unclear whether this is a causal relationship. The evaluation unit was the prefectures, so the relationship between the number of physicians and the number of nighttime emergency surgeries does not necessarily apply to each regional medical area. Second, physicians in this study refer to those in all medical specialties, which might include physicians who are not involved in surgery. The data used in this study did not include information on whether a physician was actually involved in the surgery on an individual level. Additionally, in the surveys, the names of specialties are self-reported by the physicians and may not represent their actual clinical roles, particularly their nighttime roles. As the number of physicians in each specialty showed high collinearity with one other and could not be included separately as each variable, the number of physicians in all specialties was treated as the variable. Third, as this study used Japanese data, it may not be applicable to other countries with different insurance systems.

In conclusion, the number of physicians and the number of nighttime emergency surgeries was related even after adjusting for medical supply (acute care beds), geographical factor (population density), and age structure factor (elderly population ratio). This result suggests an association between the number of physicians per population and the quality of medical services in the field of surgery.

## Supporting information

S1 File Average numbers of physicians and nighttime emergency surgeries (2015–2019). (XLSX)

S2 File Panel data of the prefectures (2015–2019). (XLSX)
